# Favorable rates of return to activity and work following lateral closing wedge distal femoral osteotomy for femoral-based symptomatic varus malalignment: an analysis at a mean 6-year follow-up

**DOI:** 10.1007/s00167-022-07303-w

**Published:** 2023-01-02

**Authors:** Marco-Christopher Rupp, Alexander Themessl, Michael Merkle, David Insam, Maximilian Hinz, Franziska L. Breulmann, Andrea Achtnich, Julian Mehl, Sebastian Siebenlist, Lukas N. Muench

**Affiliations:** 1 Department of Orthopedic Sports Medicine, Klinikum Rechts der Isar, Technical University of Munich, Ismaninger Str. 22, 81675 Munich, Germany; 2 Department of Orthopaedics and Trauma Surgery, Hospital Bogenhausen, Munich, Germany

**Keywords:** Lateral closing wedge, Distal femoral osteotomy, Varus deformity, Return to sports, Return to work, PROMs, Clinical outcomes, Functional outcomes

## Abstract

**Purpose:**

To evaluate return to sport (RTS), work (RTW) and clinical outcomes following lateral closing wedge distal femoral osteotomy (LCW-DFO) for symptomatic femoral varus malalignment.

**Methods:**

Consecutive patients who underwent LCW-DFO for symptomatic varus malalignment between 12/2007 and 03/2018 were included. The International Knee Documentation Committee (IKDC) Score, Western Ontario and McMaster Universities Osteoarthritis Index (WOMAC), Lysholm score, Tegner Activity Scale, and visual analogue scale (VAS) for pain were collected preoperatively and at a minimum of 24 months postoperatively. RTS and RTW were assessed by questionnaire.

**Results:**

Thirty-two patients (mean age: 45.9 ± 12.3 years), who underwent LCW-DFO for femoral-based varus malalignment (6.4 ± 3.0°), were included at a mean follow-up of 72.7 ± 39.1 months. The patient collective significantly improved in IKDC (51.8 ± 12.3 to 61.8 ± 21.5, *p* = 0.010; 95% CI = 3–21), WOMAC (26.7 ± 17.6 to 12.5 ± 13.5; *p* < 0.001; 95% CI = 21–6) and Lysholm (46.5 ± 19.4 to 67.9 ± 22.8 points (*p* < 0.01; 95% CI = 9–31)) scores at final follow-up. The VAS for pain reduced significantly postoperatively (4.8 ± 2.3 points to 2.6 ± 2.3 points (*p* = 0.002; 95% CI = 0–3)). Following LCW-DFO, 96% of patients returned to sports at a mean of 5.3 ± 2.9 months. Yet, a shift to lower impact sports compared to one year preoperatively was observed, with patients participating in a significantly lower number of high-impact disciplines (*p* = 0.024) and fewer hours in high-impact sports (*p* = 0.034). Twenty-three out of 24 patients returned to work at a mean 11.4 ± 10.9 weeks, with 18 patients reporting a similar or superior working ability.

**Conclusion:**

Undergoing isolated LCW-DFO for symptomatic femoral-based varus malalignment enabled the vast majority of patients to RTS and RTW along with a significant functional improvement at mid-term follow-up. However, patients’ expectations have to be adequately managed regarding a limited probability to return to high-impact sports and work after surgery.

**Level of evidence:**

Retrospective case series; Level IV.

## Introduction

In the treatment of unicompartimental knee osteoarthritis, a mounting body of evidence supports alignment corrective osteotomy aimed at unloading the affected compartment as a convincing joint preserving treatment [3, 6, 24, 34, 36, 39].

Evidence reporting on the clinical outcomes reported following corrective osteotomies [8, 15, 16, 31, 33, 34, 43] has shown significant improvement of the outcome as well as low revision rates to arthroplasty. While the reporting of patient reported outcome measures (PROMs) on a collective level is regarded as the gold standard in the scientific assessment of the outcome, these measures may often be abstract at the individual level [2]. However, subjective patient sided expectation of undergoing osteotomy may include returning preinjury lifestyles, commonly associating postoperative satisfaction with a return to preinjury sports (RTS) and work (RTW) [25]. This is especially relevant given the relatively high physical and athletic aspirations of the increasingly young patient population that is typically indicated for corrective osteotomy [15, 34]. Favorable RTS and RTW rates ranging around 94% have been reported following medial open wedge high tibial osteotomy (MOW-HTO), which historically is considered the osteotomy technique of choice for the correction of a varus malalignment [8, 33, 43].

However, an isolated tibial correction is appropriate in only 12% of the varus knees in order to avoid knee joint line (KJL) obliquity [11], which is associated with inferior clinical outcomes [18, 38, 42, 44]. While a double level osteotomy (DLO), that retains a levelled joint line, has been shown to result in favorable clinical outcomes [3, 24, 31, 34, 36], a tibial deformity is absent in up to 23% of the cases of varus malalignment, requiring an isolated femoral correction in up to 8% of the patients [11]. Yet, to date, the evidence in literature pertaining to clinical outcomes following isolated lateral closing wedge-distal femoral osteotomy (LCW-DFO) is sparse [12, 15, 21, 32]; in particular, currently there exist limited data on RTS and RTW following LCW-DFO.

Thus, the purpose of this study was to evaluate RTS and RTW as well as clinical outcomes following LCW-DFO for femoral-based symptomatic varus malalignment. It was hypothesized that undergoing a LCW-DFO would enable high RTS and RTW rates as well as satisfactory clinical outcomes at a minimum follow-up of 24 months.

## Methods

This is a retrospective monocentric outcome study of prospectively collected data including a retrospective assessment of return to sports and work. This investigation was approved by the Institutional-Review-Board (258/20S). An institutional data bank query was performed to identify patients fitting the following inclusion criteria: patients who underwent unilateral LCW-DFO for treatment of symptomatic varus malalignment between 12/2007 and 03/2018 with a minimum follow-up of 24 months. Patients were excluded if they were not available for follow-up by mail or telephone, if they underwent additional reconstructive surgery of the ipsilateral knee unrelated to the index procedure during follow-up, or if they underwent conversion to total knee arthroplasty (TKA). As previously described [31], the decision was made to exclude patients who had undergone conversion to TKA in order to assess the outcomes of patients that underwent LCW-DFO, avoiding confounding the results with data relating to subsequent reconstructive procedures. However, these patients were included in the survivorship analysis, in which survivorship was defined as not having undergone conversion to TKA or reconstructive revision surgery. Informed consent was obtained from each patient and the patients included were contacted exclusively for the purpose of this study, and parts of the subject population have been part of previous investigations at this institution.

### Patient selection

Patients were indicated for LCW-DFO if they had symptomatic varus malalignment as well as medial compartment osteoarthritis (Kellgren–Lawrence grade I–III) or medial (osteo-) chondral lesions. Contraindications for osteotomy were as following: osteochondral lesions of the lateral compartment grade 3–4 according to the International Cartilage Regeneration & Joint Preservation Society (ICRS), symptomatic patellofemoral osteoarthritis or cartilage defects, inflammatory arthropathy, lack of extension > 15° and flexion < 100°, chondrocalcinosis, chronic regional pain syndrome, or active infection.

Preoperative deformity analysis and preoperative planning was performed using one-leg standing anterior–posterior hip-knee-ankle (HKA) radiographs. The osteotomy was simulated employing the planning method according to Miniaci et al. [23] using the mediCAD® (mediCAD Hectec GmbH, Altdorf, Germany) software. Planning was performed to achieve an overcorrection of the new weight bearing line crossing the center of the tibial plateau laterally (55–65% from medial to lateral, depending on the primary pathology [10]), and the required correction (in mm) was calculated. The decision to perform LCW-DFO as opposed to a MOW-HTO was made based on the location of the deformity, as determined by the modified malalignment test as described by Paley et al. [26], with a mLDFA > 90° (with a normal mMPTA) indicating a femoral deformity.

### Surgical technique

**Fig. 1:**
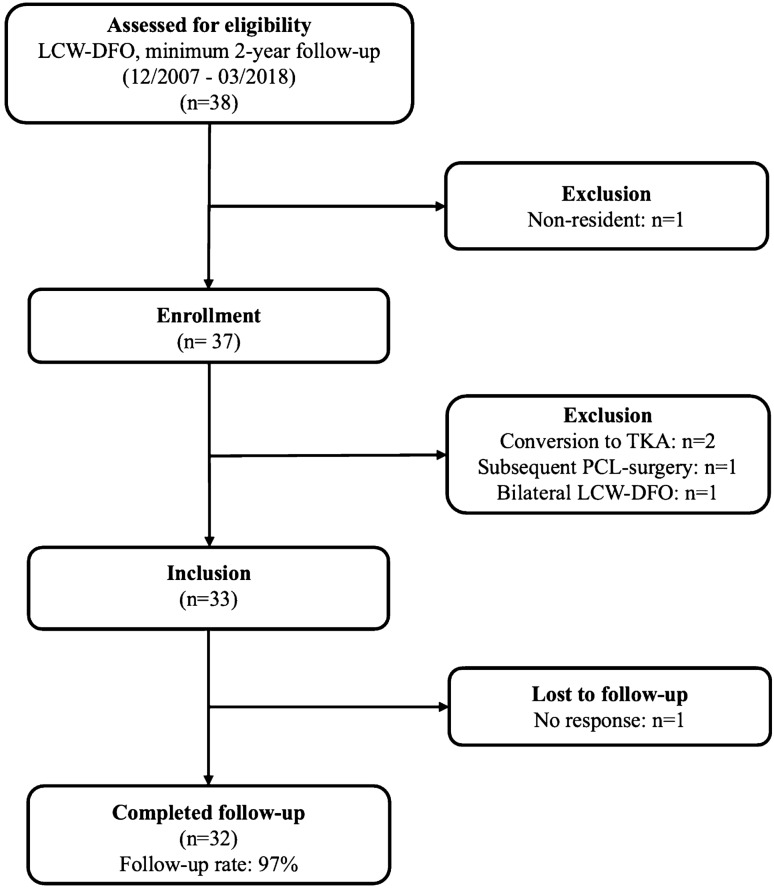
Postoperative anterior posterior radiograph of a right leg following lateral closing wedge distal femoral osteotomy for an isolated femoral-based varus malalignment secured via Tomo-Fix™ plate (DePuy Synthes, Raynham, MA, USA)

Following arthroscopy and treatment of intraarticular or ligamentous pathology, a biplanar supracondylar LCW-DFO was performed as previously described [32]. Briefly, after marking the biplanar osteotomy planes, an ascending bicortical frontal osteotomy was performed. Four axial K-wires, marking the osteotomy wedge to be excised proximally and distally, were placed for the axial osteotomy. In order to preserve the contralateral cortex, osteotomies were performed with the hinge located at a 0.5–1 cm distance from the medial cortex. The osteotomy gap was carefully closed, applying valgus stress and axial compression. The osteotomy was fixed temporarily, to control for adequate mechanical correction, and alignment was assessed via intraoperative hip-knee-ankle alignment fluoroscopy with an alignment rod [9] and adjusted as needed. The osteotomy was secured with a locking compression plate, using either a PEEK-Power™ plate (Arthrex Inc., Naples, FL, USA) or a Tomo-Fix™ plate (DePuy Synthes, Raynham, MA, USA) (Fig. 1).

### Postoperative rehabilitation

For LCW-DFO, weight bearing was limited to partial weight bearing. Following radiographic control at 6 weeks, the weight bearing was gradually increased until the patients were cleared to return to full weight bearing. At 3 months, return to sports and work was allowed for low-impact activities, and at 6 months for high-impact activity. Postoperative rehabilitation was adapted if concomitant procedures were performed at index surgery.

### Clinical evaluation

Patient reported outcome measures (PROMs) included the International Knee Documentation Committee (IKDC) Subjective Knee Form [17], Lysholm Score [22], Western Ontario and McMaster Universities Osteoarthritis Index (WOMAC), visual analogue scale for pain (VAS), and Tegner Activity Scale. The PROMs were collected preoperatively and at a minimum follow-up of two years postoperatively. Furthermore, the percentage of patients surpassing the minimally clinically important difference (MCID) for WOMAC score [19], IKDC subjective knee form [28] and Lysholm Score [5] was calculated for the patients mathematically able to reach MCID postoperatively (WOMAC > 16.1; IKDC < 88; Lysholm < 90). The outcome scores as well as the RTS/RTW questionnaires were provided by mail and analyzed by orthopedic sports medicine physicians in training (MCR, AT). Furthermore, complications requiring revision surgery were collected both by chart review and questionnaire.

### Return to sports

To evaluate return to sports (RTS), a previously developed questionnaire to assess RTS following alignment corrective osteotomy was administered [31]. Patients reported their specific preoperative (one year prior to the osteotomy) and postoperative (final follow-up) participation in 34 different sporting activities. Parameters included the level of sport, frequency of participation and duration of each session. The types of sports were categorized by low, intermediate, high impact [40], and the timing of RTS, timing of return to the current level of sports at final follow-up and qualitative change of sporting ability were evaluated. The reasons for restrictions in postoperative sporting activities were differentiated by additional questions investigating the reason for deterioration (due to the operated knee, fear of reinjury, other physical problems not related to the operated knee, or due to non-physical personal reasons such as shortage of time due to obligations in family, professional career.). Finally, patients were asked to rate their current function of the leg (excellent, good, satisfactory, bad) and indicate, if they had received further surgery.

### Return to work

Similarly, a previously developed questionnaire to assess RTW following alignment corrective osteotomy was administered [31]. In summary, the patients’ occupation (employment, self-employed, housework, retired, unemployed) and working hours per week (0, 0–10, 10–20, 20–30, 30–40, > 40) both prior to surgery and one year postoperatively were asked. Patients were questioned on the physical strain of their occupation according to the classification of the REFA association (occupation without specific physical strain or with either small, moderate, hard or most heavy physical strain, defined by specific criteria) prior to surgery and one-year postoperatively, as previously published in the setting of osteotomy [35]. Time of sick leave as well as the time of RTW and recovery to full current physical working ability were quantified. Finally, the qualitative change of working ability (ordinal scale consisting of “improved,” “equal to preoperative state,” or “deteriorated”) was assessed. For both RTS and RTW questionnaires, completion according to the instructions was a precondition for inclusion into the final analysis; two attempts to contact the patient via telephone for clarification were made.

### Statistical analysis

A total sample size of 14 subjects to detect the minimal clinically important difference of the WOMAC score of 16.1 points[19] and a standard deviation of 10 points in order to achieve a statistical power of 0.8 was determined in an a priori power analysis, performed with G*Power (Erdfelder, Faul, Buchner, Lang, HHU Düsseldorf, Düsseldorf, Germany).

Categorical variables were reported as count and percentages. Continuous variables were reported as mean ± standard deviation. The Shapiro–Wilk-Test was employed to determine the distribution of continuous variables. The parametric paired t-test or the nonparametric Wilcoxon-test for two related samples was used to compare pre- and postoperative continuous parameters, while the McNemar test or the sign-test was applied for pre-to postoperative comparisons of categorial parameters as statistically appropriate. The level of significance was set at *p* < 0.05. 95% confidence intervals were calculated. Statistical analysis was performed using SPSS software version 26.0 (IBM-SPSS, New York, USA).

## Results

**Fig. 2:**
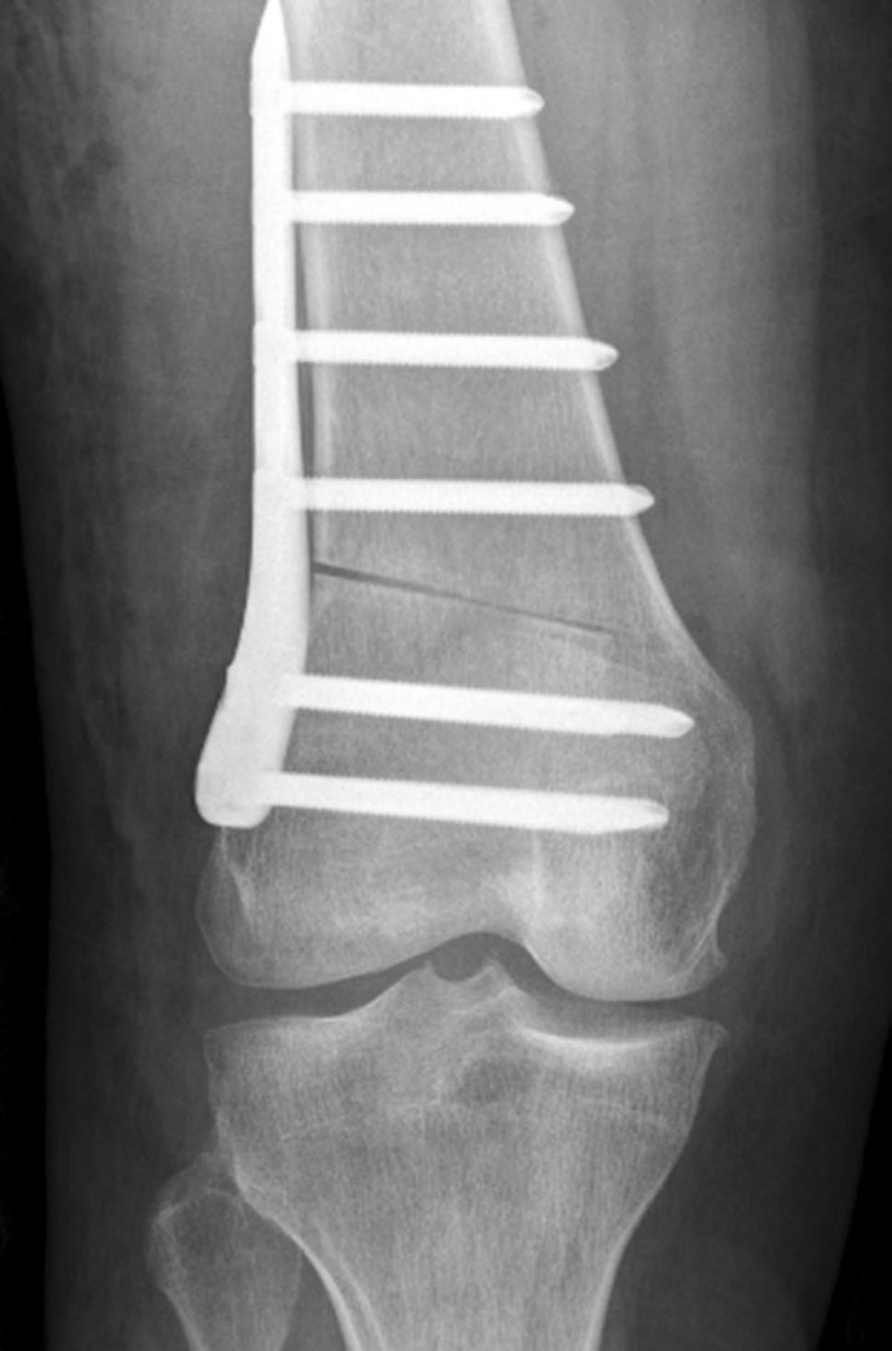
Flowchart visualizing the patient population for this study after accounting for inclusion criteria, exclusion criteria, failures and those lost to follow-up. LCW-DFO; lateral closing wedge distal femoral osteotomy, *TKA* total knee arthroplasty, *PCL* posterior cruciate ligament

A review of the institutional database identified 37 patients who underwent LCW-DFO with a minimum 24-months follow-up between 12/2007 and 03/2018. The process of inclusion and exclusion is detailed in Fig. 2. After the application of exclusion criteria (conversion to TKA: *n* = 2, bilateral LCW-DFO: *n* = 1; subsequent PCL reconstruction unrelated to index surgery following a motor vehicle accident: *n* = 1), 33 patients were included in the final study population. Despite best efforts to attain follow-up, one patient could not be reached for follow-up evaluation and was considered lost to follow-up. Therefore, final data analysis was available for 32 patients (18 men, 14 women; 97% follow-up). Of those, 27 patients chose to participate in the RTS/RTW survey (84%). Comprehensive information on the demographic and surgical data is demonstrated in Table 1.

**Table 1:** Demographic and surgical characteristics of the study cohort

Variable	Total study group
Patients*, n*	32
Sex	
Female*, n (%)*	14 (44%)
Male*, n (%)*	18 (56%)
Age^a^ (years)	45.9 ± 12.3 (18–72)
Laterality	
Left*, n (%)*	16 (50%)
Right*, n (%)*	16 (50%)
BMI (kg/m^2^)*	29.0 ± 5.0 (18–45)
Smoker	
Yes*, n (%)*	9 (28%)
No*, n (%)*	16 (50%)
N.a.*, n (%)*	7 (22%)
Preoperative mechanical knee angles	
mMPTA, °	88.7 ± 2.2 (86–93)
mLDFA, °	92.6 ± 2.1 (90–98)
JLCA, °	2.2 ± 1.7 (0–6)
mFTA, °	6.4 ± 3.0 (2–13)
Implant used	
Tomo-Fix*, n (%)*	27 (84%)
Peek Power Plate*, n (%)*	5 (16%)
Indications/Main indication for surgery	
Medial compartment osteoarthritis, *n (%)*	25 (78%)
Osteochondral lesion, *n (%)*	6 (19%)
Medial compartment overload, *n (%)*	1 (3%)
Concomitant pathologies	
Meniscal tear, *n (%)*	8 (25%)
ACL/PCL insufficiency, *n (%)*	4 (13%)
Patellofemoral chondral defect, *n (%)*	4 (13%)
Concomitant procedures^c^	
OATS, *n (%)*	2 (6%)
Partial meniscectomy, *n (%)*	7 (22%)
Meniscus repair, *n (%)*	1 (3%)
ACL reconstruction*, n (%)*	1 (3%)
Previous procedures	14 (44%)
Meniscus surgery, *n (%)*	9 (28%)
Microfracture, *n (%)*	2 (6%)
ACL ligament-reconstruction, *n (%)*	4 (13%)

Continuous variables are presented as mean ± standard deviation (range); Categorical variables are presented as count and percentage

*BMI* body-mass-index, *n.a.*, not available; *mLDFA*, mechanical lateral distal femur angle, *mMPTA*, mechanical medial proximal tibia angle; *JLCA*, joint line convergence angle; *mFTA*, mechanical femorotibial angle; *ACL*, anterior cruciate ligament; *PCL*, posterior cruciate ligament; *OATS* osteochondral autograft transfer system

^a^Age at surgery

^b^Medial or lateral meniscus, partial resection or suture

*Data available for *n* = 27 patients

### Clinical outcome

Overall survivorship at a final follow-up of 72.7 ± 39.1 months was 94%. The IKDC significantly increased from 51.8 ± 12.3 to 61.8 ± 21.5 (*p* = 0.10; 95% CI = 3–21), the WOMAC score significantly improved from 26.7 ± 17.6 to 12.5 ± 13.5 (*p* < 0.001; 95% CI = 21–6), and the Lysholm score significantly increased from 46.5 ± 19.4 to 67.9 ± 22.8 points (*p* < 0.01; CI = 9–31). The pain intensity assessed with the VAS pain scale significantly declined from 4.8 ± 2.3 points to 2.6 ± 2.3 points (*p* = 0.002; 95% CI = 0–3), while the Tegner activity scale did not change significantly, with 3.9 ± 2.6 points compared to 3.5 ± 1.7 points (p = n.s.). In total, 73% of the patients surpassed the MCID in one of the functional knee scores administered, with 62% of the patients surpassing the MCID for WOMAC, 52% for the IKDC and 68% for the Lysholm score.

### Return to sports

**Fig. 3:**
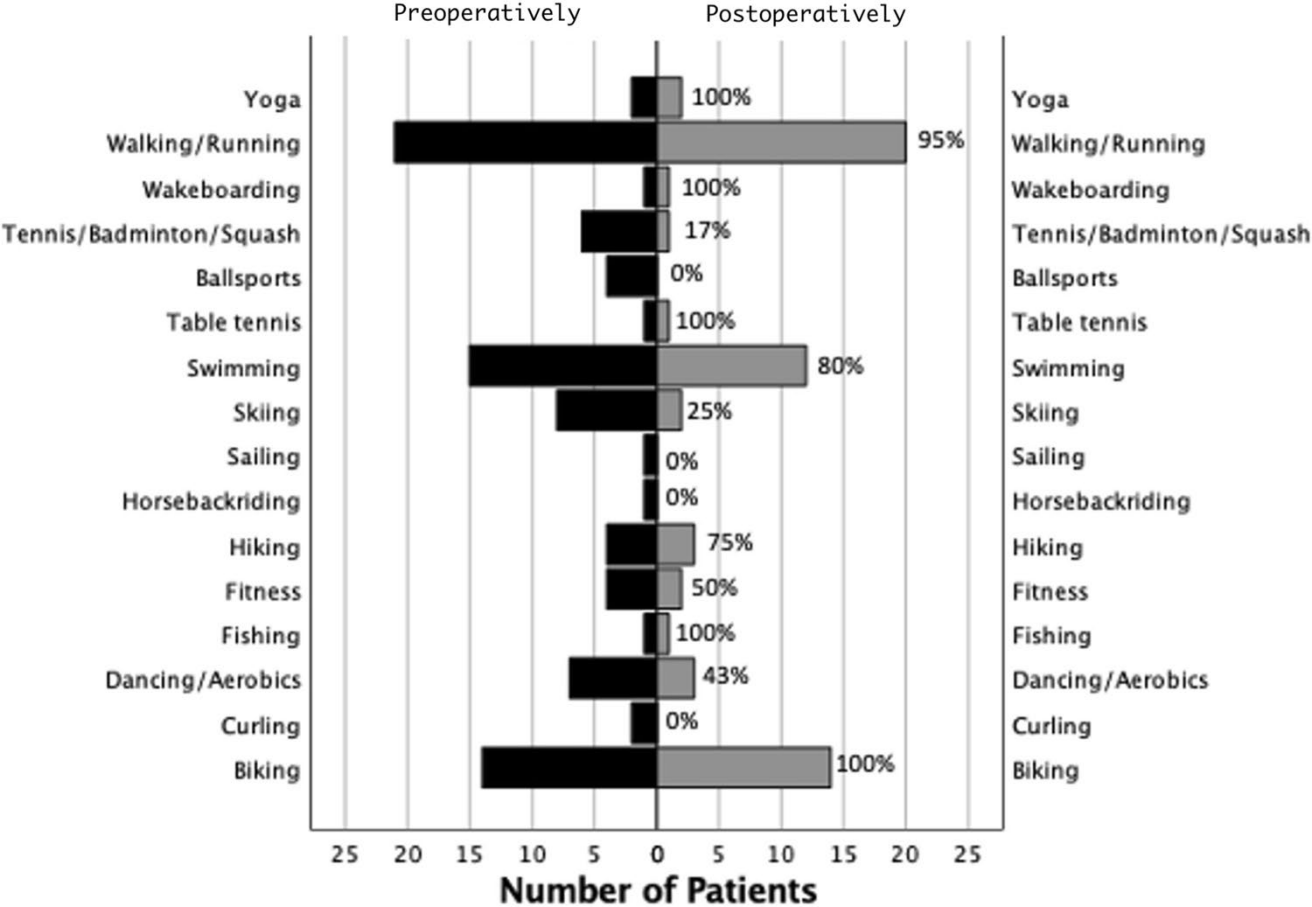
Histogram depicting the sport-specific activities of the patient population pre- and postoperatively. Only disciplines, that were practiced by at least one patient pre- or postoperatively were included into the diagram. The category “ballsports” is summarizing soccer, volleyball and basketball

Preoperatively, 22 patients participated in one or more sports at a predominantly recreational level (82%). Of those, 21 (96%) had returned to sports postoperatively. One patient not participating in sports preoperatively had started to participate in sports following surgery. Patients returned to sports at 5.3 ± 2.9 months, while the current level was reached at 11.0 ± 9.8 months. Across the study population, compared to one year preoperatively, neither the number of disciplines (2.2 ± 2.7 vs. 1.7 ± 2.0, *p* = n.s.), nor the hours per week (9.8 ± 9.9 vs 9.6 ± 9.8, *p* = n.s.) had significantly changed at final follow-up. Regarding sports intensity, patients involved in sports participated in a significantly lower number of high-impact disciplines and fewer hours in high-impact sports compared to one year preoperatively, while these numbers did not change significantly for intermediate and low-impact sports; details can be found in Table 2. Information on return to specific disciplines can be found in Fig. 3.

**Table 2:** Athletic activity stratified by impact pre- and postoperatively

Sports disciplines	Preoperatively	Postoperatively	*P* value
High impact			
Patients, *n* (%)	7 (26%)	2 (7%)	n.s
Number of disciplines^a^	0.4 ± 0.9	0.1. ± 0.4	0.024*
Hours per week^a^	1.3 ± 2.6	0.2 ± 0.8	0.034*
Intermediate impact			
Patients, *n* (%)	9 (33%)	7 (26%)	n.s
Number of disciplines ^a^	0.4 ± 0.6	0.3 ± 0.6	n.s
Hours per week^a^	1.3 ± 2.8	2.2 ± 7.3	n.s
Low impact			
Patients, *n* (%)	21 (78%)	22 (82%)	n.s
Number of disciplines ^a^	2.2 ± 1.9	1.9 ± 1.4	n.s
Hours per week^a^	7.2 ± 6.8	7.2 ± 7.1	n.s

Continuous variables are presented as mean ± standard deviation; Categorical variables are presented as count and percentage; analysis of the study population included in the return to sports analysis (*n* = 27)

^a^Number represents the mean ± standard deviation of the active study population

^*^Statistically significant difference between pre- and postoperative data (level of significance, *p* < 0.05)

Regarding subjective function of the knee, a total of 4 patients (15%) reported their leg function to be “excellent,” while 10 patients (37%) classified it as “good,” 7 patients (26%) as “satisfactory” and 6 patients (22%) as “bad.” A total of 12 patients (46%) reported a subjectively “improved,” 6 patients (23%) an “equal” and 8 patients (31%) a “worse” ability to compete in sports and participate in their activities. Of the patients that indicated a “worse” subjective satisfaction in their activity, 7 patients (88%) attributed the deterioration to the operated knee and 1 patient to a medical reason not associated with the surgery.

### Return to work

Within the study population, 24 (89%) patients reported working preoperatively. Postoperatively, 23 (96%) returned to work, and 1 patient (4%) reported to be unemployed*.* RTW was possible at a mean of 11.4 ± 10.9 weeks, while regaining full current physical working ability was achieved at a mean of 5.4 ± 4.0 months. Preoperatively, 16 patients (59%) had indicated a high physical strain in work, pursuing an occupation with moderate to most heavy load postoperatively. Postoperatively, 15 patients (94%) were able to return to this work intensity; more detailed data can be found in Table 3. While 10 patients (37%) reported an “improved” and 8 patients (30%) an “equal” working ability, 9 patients (33%) reported a “worse” working ability following surgery. Of those, 3 patients (33%) who reported a worse working ability indicated reasons other than the operated leg.

**Table 3:** Occupational activity pre- and postoperatively

Variable	Preoperatively	Postoperatively	p value
Type of work			n.s
Employed, *n* (%)	18 (67%)	17 (63%)	
Self-employed, n (%)	4 (15%)	4 (15%)	
Housework, *n* (%)	2 (7%)	2 (7%)	
Retired, *n* (%)	3 (11%)	3 (11%)	
Unemployed, *n* (%)	–	1 (4%)	
Physical load^a^			n.s
Without specific strain, *n* (%)	4 (15%)	6 (22%)	
Small strain, *n* (%)	7 (26%)	6 (22%)	
Moderate strain, *n* (%)	8 (30%)	9 (33%)	
Hard strain, *n* (%)	4 (15%)	4 (15%)	
Most heavy strain, *n* (%)	4 (15%)	2 (7%)	
Working hours			n.s
0 h	3 (11%)	5 (19%)	
0–10 h	3 (11%)	3 (11%)	
10–20 h	3 (11%)	5 (19%)	
20–30 h	2 (7%)	–	
30–40 h	6 (22%)	6 (22%)	
> 40 h	10 (37%)	8 (30%)	

Categorical variables are presented as count and percentage; analysis of the study population included in the return to work analysis (*n* = 27)

## Discussion

The most important finding of this study was that patients undergoing LCW-DFO for femoral-based varus malalignment reported high RTS and RTW rates as well as clinical improvement at an average mid-term follow-up of 6 years postoperatively. These findings may be helpful managing expectations for sports- and work-related outcomes after LCW-DFO.

The prevalence of a mLDFA of 92.6 ± 2.1° and mean mMPTA of 88.7 ± 2.2° within in the patient population of the present study acknowledges the presence of a patient subgroup with a predominantly femoral deformity, as suggested by previous studies [11, 27]. Previous radiologic reports on this patient collective, which showed that the mean tibial KJL is already tilted laterally 2.2° at a mean mMPTA of 86.4 ± 2.4° [27], support to not further increase lateral tibial KJL tilt by a MOW-HTO to avoid exceeding the postoperative threshold of 4° KJL obliquity [38]. Tibial overcorrection resulting in more than 4° of postoperative KJL obliquity results in lateral compartment pain [18] and inferior patient reported outcomes [38]. The biomechanical correlate of these inferior outcomes in the setting of an oblique KJL includes supraphysiological articular contact pressure [44], tibial subluxation [44], subsequent detrimental effects on tibiofemoral instability [13], and ultimately progression to compartmental osteoarthritis [38]. These observations strengthen the rationale to perform an isolated femoral correction in patients with isolated femoral varus deformity [11].

While the reporting of clinical outcome data following isolated LCW-DFO has only been limited to small case series [12, 15, 21, 32], the results of the present study underscore the positive outcomes following LCW-DFO. The survivorship of 94% at a mean follow-up of 72.7 ± 39.1 months in the present study is in line with previous data published following LCW-DFO, ranging around 94% at 5 years, as well as MOW-HTO, ranging around 90–99% at mid-term follow-up [7].

Furthermore, the clinical outcome as measured by PROMs reported in the present study confirms the results of previous case series, with Lysholm scores ranging around 68 points [15] and transformed WOMAC scores ranging around 80 ± 20 points [41]. The level of pre- to postoperative improvement of 21.4 points in the Lysholm score in the present study is similar compared to 19.6 points that was previously reported [12]. Furthermore, the PROMs reported following LCW-DFO in the present study are comparable to outcomes following isolated MOW-HTO, in which the mean postoperative Lysholm score is ranging between 67 and 76 points [4, 20] and IKDC score is ranging between 67 and 69 points at a comparable follow-up period [4, 37].

Regarding sports-related outcomes, the RTS rate of 96% following LCW-DFO was high and similar to other osteotomies used in the correction of varus malalignment. More specifically, for MOW-HTO, pooled RTS rates were reported to range around 94% [16], while for DLO, RTS rates as high as 90% to 96% were observed [31, 34]. However, comparable to previous reports in HTO [16], DFO [15] and DLO [31], RTS was limited in regards to the level of activity as well as the types of activity the patients returned to in the present collective. With only 7% of the patients involved in high-impact sports following LCW-DFO in this study compared to 28% preoperatively, this finding is in accordance with previously reported rates of return to high-impact activity following MOW-HTO [16] and DFO [15] and DLO [30], ranging around 9%, 6% and 23%, respectively. Of note, the collective in the present study did not improve in their average Tegner activity scale scoring. With returning to their sports at a mean of 5.3 ± 2.9 months following LCW-DFO, the timeframe required to RTS is comparable to MOW-HTO, with 75% [14], as well as DFO, with 71% of the patients returning to sports within 6 months [15], but substantially shorter than DLO, with a RTS-rate of 7.2 ± 4.9 months [30].

When evaluating RTW, 96% of the patients working preoperatively were able to RTW postoperatively following LCW-DFO. This is in the range with previously reported RTW-rates after DFO (91%) [15], MOW-HTO (72%- 94%) [8, 33, 43] and DLO (92%)[30]. Following a similar trend as the high-impact sports related outcomes, and comparable to observations following HTO [1] and DFO [29], only 50% of the patients involved in the most strenuous labor preoperatively had to discontinue their work following LCW-DFO. With the patients returning to their work at a mean of 11.4 ± 10.9 weeks following LCW-DFO, the timeframe for RTW was comparable to isolated HTO, with 10 and 22 weeks [1, 4, 8, 33, 35], but shorter compared to DLO, with RTW time frames of 6 ± 9 months [30].

In summary, these data may be helpful in preoperatively managing patients’ expectations regarding recovery as well as sports- and work-related outcomes after LCW-DFO, as the procedure was demonstrated to effectively return patients to athletic and professional activity at a low complication rate at a mean 6 year follow-up.

There were several limitations to the study. First, while representative of the patient population indicated for LCW-DFO, the heterogeneity of the study population regarding concomitant pathologies and procedures may have biased the outcomes. Second, excluding *n* = 2 patients converted to TKA at final follow-up, in an attempt to adequately reflect the RTS- and RTW-related outcomes comparable to previous studies [30], may have skewed the results. Third, due to the absence of validated RTS questionnaires in the setting of lower extremity alignment correction, non-validated questionnaires designed based on previous studies for knee osteotomy [1, 14, 15, 29, 30] were used for qualitative analysis of the RTS and RTW. Fourth, postoperative radiological outcomes were beyond the scope of this study and thus not reported. Sixth, the study inherits the associated biases of a retrospective case series design not including a comparative analysis to DLO or HTO. Last, the external validity of the results may be limited due to the monocentric study design in a single reference center for lower extremity osteotomies.

## Conclusion

Undergoing isolated LCW-DFO for symptomatic femoral-based varus malalignment enabled the vast majority of patients to RTS and RTW along with a significant functional improvement at mid-term follow-up. However, patients’ expectations have to be adequately managed regarding a limited probability to return to high-impact sports and work after this surgery.

## Data Availability

The participants of this study did not give written consent for their data to be shared publicly. As such, due to the sensitive nature of the research, supporting data is not available.

## Abbreviations

DFO: Distal femoral osteotomy
DLO: Double level osteotomy
HTO: High tibial osteotomy
IKDC: International Knee Documentation Committee
IRCS: International Cartilage Regeneration & Joint Preservation Society
LCW: Lateral closing wedge
MCID: Minimally clinically important difference
mLDFA: Mechanical lateral distal femur angle
mMPTA: Mechanical medial proximal tibia angle
MOW: Medial open wedge
PROM: Patient reported outcome measure
RTS: Return to sport
RTW: Return to work
TKA: Total knee arthroplasty
VAS: Visual analogue scale
WOMAC: Western Ontario and McMaster Universities Osteoarthritis Index
